# 120 Years of U.S. Residential Housing Stock and Floor Space

**DOI:** 10.1371/journal.pone.0134135

**Published:** 2015-08-11

**Authors:** Maria Cecilia P. Moura, Steven J. Smith, David B. Belzer

**Affiliations:** 1 Joint Global Change Research Institute, Pacific Northwest National Laboratory, College Park, Maryland, United States of America; 2 Department of Atmospheric and Oceanic Science, University of Maryland, College Park, Maryland, United States of America; 3 Pacific Northwest National Laboratory, Richland, Washington, United States of America; East China University of Science and Technology, CHINA

## Abstract

Residential buildings are a key driver of energy consumption and also impact transportation and land-use. Energy consumption in the residential sector accounts for one-fifth of total U.S. energy consumption and energy-related CO_2_ emissions, with floor space a major driver of building energy demands. In this work a consistent, vintage-disaggregated, annual long-term series of U.S. housing stock and residential floor space for 1891–2010 is presented. An attempt was made to minimize the effects of the incompleteness and inconsistencies present in the national housing survey data. Over the 1891–2010 period, floor space increased almost tenfold, from approximately 24,700 to 235,150 million square feet, corresponding to a doubling of floor space per capita from approximately 400 to 800 square feet. While population increased five times over the period, a 50% decrease in household size contributed towards a tenfold increase in the number of housing units and floor space, while average floor space per unit remains surprisingly constant, as a result of housing retirement dynamics. In the last 30 years, however, these trends appear to be changing, as household size shows signs of leveling off, or even increasing again, while average floor space per unit has been increasing. GDP and total floor space show a remarkably constant growth trend over the period and total residential sector primary energy consumption and floor space show a similar growth trend over the last 60 years, decoupling only within the last decade.

## Introduction

From 1950 to 2011 primary energy consumption in the U.S. residential sector increased from 5,989 to 21,411 trillion BTU, accounting for approximately one-fifth of the country’s total primary energy consumption and energy-related CO2 emissions [1]. The size and location of residential buildings are key drivers of energy consumption and environmental impacts. The spatial structure of residential neighborhoods, such as suburbanization vs urbanization, directly impacts land-use change, and influences transportation patterns. The size of residential buildings, particularly their floor space area, is a direct driver of energy demands and associated environmental impacts.

According to many studies, the residential sector has one of the most significant cost-effective potentials for reducing energy-related greenhouse gas (GHG) emissions [2], [3]. Residential and commercial buildings are also one of the key drivers of seasonal and diurnal changes in electricity demand. A wide range of residential energy consumption drivers, including socio-economic, environmental, household and structural indicators, have been investigated in the literature using a variety of methodologies [4–8], but the importance of floor space as a main driver of energy consumption stands out in many studies.

Kelly [8] examines direct and indirect residential energy drivers in the English residential sector and shows that household size has the largest effect on energy consumption, followed closely by floor space and household income. An analysis by Clune et al. [7], motivated by the fact that Australian houses have been increasing in size, develops a residential GHG emissions calculator to investigate the capacity of building codes to meet emission reduction targets, and shows that house size has a major impact on the thermal performance. Tso and Guan [4] develop a multi-regression model which quantifies the explanatory power of residential energy consumption indicators and differentiates regional from household characteristics effects. They estimate that the expected annual household energy consumption increases by 488,791 kWh as average house size increases by one square foot from regional averages, and that single-family detached homes consume the most energy in all regions. Kavousian et al. [6] develop two separate models to analyze daily electricity maximums and minimums and examine four main factors that affect residential electricity consumption: weather and location; physical characteristics of the building; appliance and electronics stock; and occupancy and occupants’ behavior towards energy consumption. The study concludes that external conditions based on region have the largest effect on electricity use, explaining up to 46% in the consumption variability. Physical characteristics of housing are the next most important factor, with house size being the most significant of all physical characteristics (house size determines 21% of the variability in winter minimum consumption), while house age and type of building are less significant.

The residential sector has been referred to as an “undefined energy sink” [9], [10], partially because of a lack of complete and reliable datasets. The main purpose of this paper is to address two main limitations which exist in the floor space data available for U.S. residential sector research. Neither of these issues has been previously addressed in the literature.

The first contribution of this work is the development of a *long-term* residential floor space time-series for the U.S., for the 120-year period 1891–2010, which addresses the first limitation encountered in the datasets: the temporal scope of available data. With the technological advancements first made possible by the Industrial Revolution, the manner in which societies consume energy is constantly changing, and long-term data sets are of fundamental importance. The development of a historical floor space series going back one century or more broadens the analysis perspective and allows energy consumption patterns to emerge that might otherwise be difficult to identify.

For the U.S., no floor space estimates have been published for longer-term analysis of residential energy consumption drivers. Partial census data for housing units exist for the late 19th and early 20th century, but this data have not yet been translated into floor space. A recent report developed energy efficiency indicators for the U.S. economic sectors and focused on floor space as the main measure of the evolution of these indicators for the residential sector. The report developed housing stock and floor space time-series based on Residential Energy Consumption Survey (RECS) and the American Housing Survey (AHS), but these time series were limited to the 1970–2011 period. The report points out, “there are no publicly available annual time series estimates of residential floor area in the U.S.” [11]. Another recent study estimated historical physical stocks of 91 in-use products in the U.S., including residential and commercial building floor space for 1950–2009. Residential floors pace trends were based on RECS over 1979–2009, with earlier trends extrapolated using commercial floor space estimates from PNNL [12]. In one of the few long-term studies of energy services, Fouquet (2014) developed a time series from 1700 to 2010 for the consumption of energy services in the U.K., including domestic heating and lighting, but not including floor space [13]. Aside from these examples, the studies we examined are based on energy and floor space datasets that span at most three decades.

The second contribution of this work addresses another limitation in the available datasets. C*onsistent* data for energy consumption by end-use service are not readily available. There are many barriers to effective data collection in the residential sector, such as the complexity of structural characteristics of buildings, privacy issues, and the fact that occupant behavior varies widely and can impact energy consumption [10]. Because of this central barrier, studies on the residential sector have been hindered by insufficient or inconsistent information. In spite of the strong potential for emissions mitigation in the residential sector, there are more available data on energy consumption drivers in the industrial, agricultural and transportation sectors. Centralized ownership and high levels of regulation in the latter sectors, for instance, facilitate access to data sources [10], [14], [15]. This limitation in available data extends to housing stock and floor space data. As an example, even for the last 30 years there are many inconsistencies in floor space data from different sources, as also discussed in more detail below.

These two main limitations in the literature were addressed by first developing an estimate of housing units, disaggregated over vintage (groups of housing units built in the same period), based on national housing surveys conducted by the U.S. Census Bureau. These surveys report partial vintage-disaggregated datasets, but no studies have combined these datasets to develop consistent long-term estimates of the number of housing units in each vintage group. Housing stock estimates were then combined with estimates of average floor space per housing unit by vintage, and the corresponding floor space time series were obtained, also disaggregated by vintage, for three building types: single-family, multi-family and manufactured homes.

Section 2.1 provides an overview of the survey data used in this work. The methodologies employed in the various stages of development of the time-series are addressed in Sections 2.2–2.5. Section 3 presents the resulting time-series and examines the relationships between the long-term evolution of U.S. floor space and three indicators, namely population, number of housing units and household size. We also compare the evolution of floor space with the evolution of GDP and of primary energy consumption. The final section presents a conclusion and suggestions for future research.

## Data and Methodology

### Background on data and methodology

The data used in this work are derived from the U.S. Census Bureau and the U.S. Department of Housing and Urban Development. National survey data for detached and attached single-family homes, multi-family homes and manufactured homes were taken into account. Data included the number of new constructions, housing stock (the total number of housing units), the number of housing units by vintage (building age), and the average floor space for each building vintage and type.

The U.S. Census Bureau Decennial Census has been conducted since 1790 and has evolved considerably over time. The Decennial Census uses what is known as a “short form questionnaire”, where a limited number of basic questions are asked of the entire population and every housing unit in the country. One of the most significant innovations occurred in 1940, when statistical sampling was first introduced. As part of this new methodology, the Decennial Census started collecting additional questions from a small rotating sample of the population and housing units. These surveys, referred to as the “long form questionnaire”, collected more detailed data related to housing than the short-form questionnaire. This methodology innovation is reflected in the better quality of the post-1940 survey data, leading to greater accuracy in the last seven decades of the various time-series that were developed in this work.

In collaboration with the Department of Housing and Urban Development, in 1973 the U.S. Census Bureau started conducting a national Annual Housing Survey, with sampling sizes of 55,000 units. In this longitudinal survey, the same samples were followed annually until 1981, when surveys began to be conducted only on odd-numbered years. After 1983, the Annual Housing Survey became the American Housing Survey, which is still conducting surveys today, with larger sample sizes.

The overall estimation method employed to develop the housing stock and floor space series was a bottom-up approach designed to minimize the effect of data inconsistency and incompleteness in the survey data. Datasets from different sources and periods were adjusted and combined on a case-by-case basis. Whenever possible, approaches were chosen which made it possible to make judgments about the quality of the data, as in the case of the inventory modeling approach, discussed in Section 2.3. The main assumptions made to address these data issues and produce consistent housing stock and floor space estimate are discussed in the following four sections.

### Estimation of construction and housing stock time-series

The first stage in the estimation of the floor space time-series consisted in the development of independent construction and stock time-series from survey data. Construction data were compiled based on several U.S. Census Bureau survey datasets: historical statistics for the first 80 years (1891–1970), new residential construction data for the next 40 years (1971–2010), and manufactured homes shipment surveys for 1959–2010 (S2 File). Stock data were based on U.S. Census Bureau Statistical Abstracts for 1900–2002, on U.S. Census Bureau Census of Housing for 1940–2000, on U.S. Department of Housing and Urban Development Housing Trends for 1973–1989 and on U.S. Census Bureau Annual Housing Survey for 1973–1983, as well as on American Housing Survey data for 1991–2011 (S3 File).

Some of the issues encountered in the survey datasets included data subject to large gaps, irregular classification aggregation and various inconsistencies and unexpected data patterns. Missing data were interpolated or extrapolated. For instance, data for manufactured homes, in the form of the number of shipments, is only available starting in 1947, and stock data is not available for 1891–1899 (S2 and S3 Files). As examples of irregular aggregation, construction data do not always include data on farms, and 1889–1899 construction data are not disaggregated by building type (S2 File). Methodological changes possibly associated with the re-structuring of the Annual Housing Survey resulted in 1973–1983 stock data that are not consistent with data reported after 1985 (S3 File). Further inconsistencies resulted from data related to occupied versus vacant floor space, which were resolved by taking into account data for all floor space.

**Fig 1:**
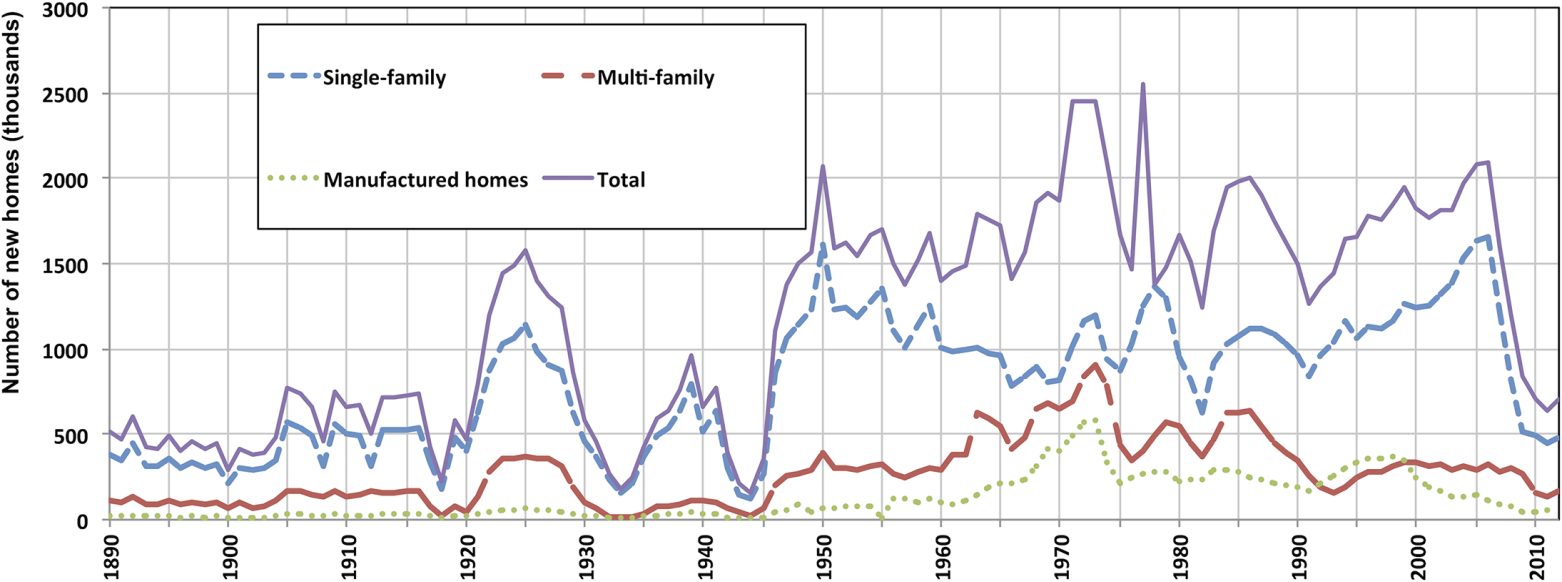
Estimation of new housing completions (construction) of 3 building types, 1891–2010. Sources: Compiled from Historical Statistics of the United States, Colonial Times to 1970—Part 2; U.S Census Bureau, New Residential Construction; Manufactured Homes Survey (S2 File).

Our resulting construction is shown in Fig 1. The stock time-series is shown in the Results and Discussion section.

### Estimation of annual retirement time-series: the inventory modeling approach

The new construction and survey stock data described in the previous section was then used to develop an annual time-series of retiring housing stock, as will be shown below. A simple inventory modeling approach was employed for this purpose, similar to the approach used in a study on U.S. energy intensity indicators, where incomplete and inconsistent American Housing Survey data is used to develop a smooth U.S. housing unit time-series for 1985–2011 [11].

Housing stock *U*_*t*_ for year *t* consists of the stock *U*_*t-1*_ corresponding to the previous year, to which new construction (*C*_*t*_) and housing retirements (*R*_*t*_) are respectively added and subtracted. This relationship, described in Eq 1, is effectively a ‘stock or housing unit conservation equation’, as it describes the evolution of the housing units over time. It should be noted that the annual retirement series *R*_*t*_, which results in an aggregate estimate, across all vintages, of retiring building stock in a particular year *t*. In the next section, the approach used to disaggregate retirement time-series by vintage is described.

$$U_{t}=U_{t-1}+C_{t}-R_{t}$$ Eq 1

The main advantage of this approach is its simplicity, particularly in light of the large uncertainty in the data. Another advantage of this approach is that it provides a measure of the quality of the survey data. The inventory modeling approach allowed us to identify inconsistencies in the survey data and was helpful in devising strategies to minimize these inconsistencies. For example, when the conservation equation is applied to the new construction and survey stock data for certain years, the number of new construction in a particular year does not always account for the increase in stock from one year to the next. The retirement series obtained directly from the conservation equation are shown in S1 Fig (for single- and multi-family units, see dotted lines).

To address a possible under-reporting of construction, different approaches were employed for the three building types. Stock data appeared to be more reliable than construction data for all building types and were chosen as a priority dataset. The approaches described below therefore preserved stock data, and any necessary adjustments were generally made to construction data and the corresponding retirement time-series (to leave stock data unaltered, adjustments to the construction data implied an equivalent adjustment to the retirement time-series, and vice-versa). The adjusted retirement time-series for all three building types are shown in S1 Fig

**Fig 2:**
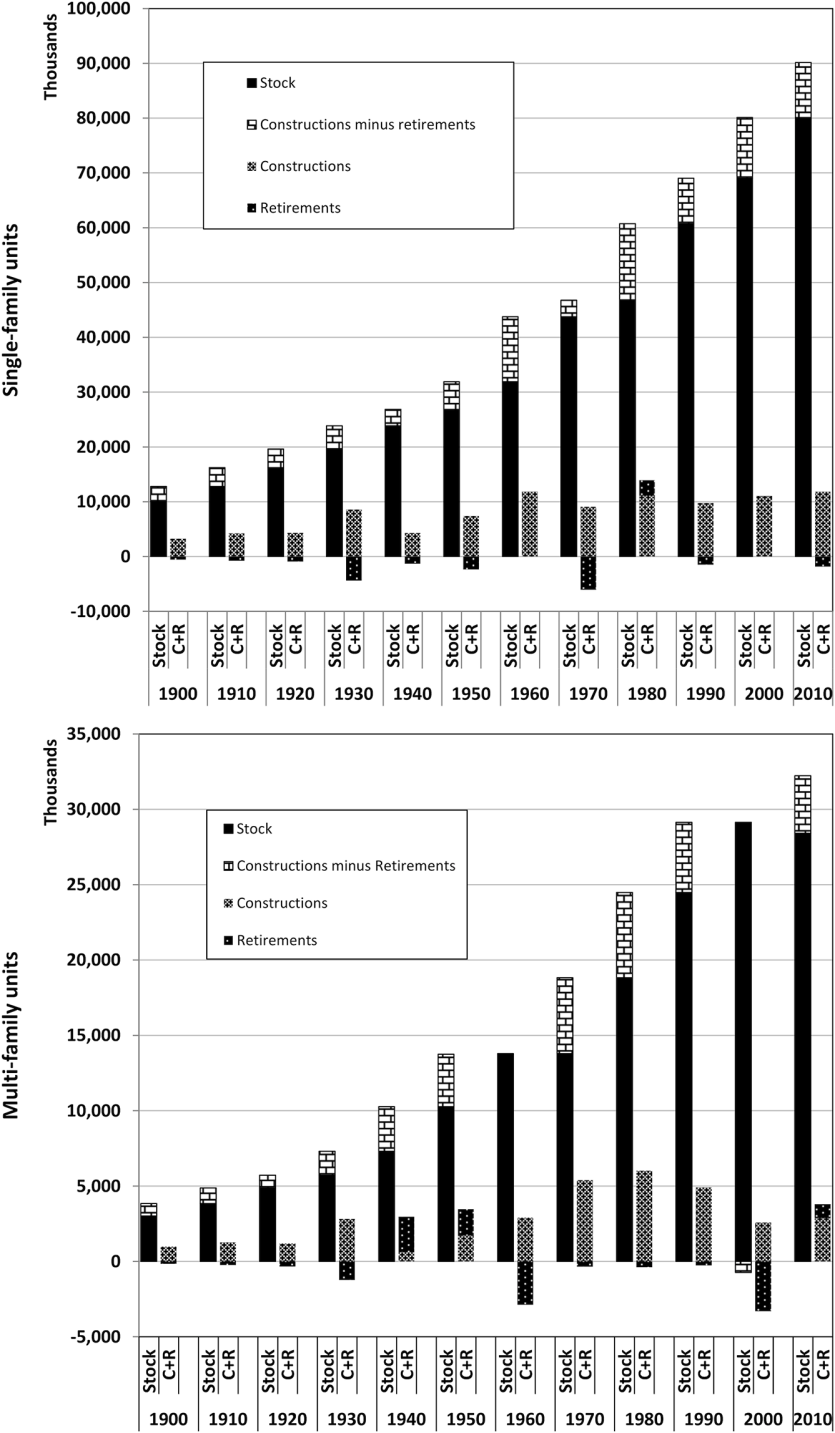
Decadal stock conservation based on survey stock data, 1891–2010, for single- and multi-family units. As an example, in the single-family graph, consider the pair of columns labeled 1930: the left column shows the total stock in 1930 subdivided into the total stock in 1921 and the net stock addition in 1921–1930. The right column shows the 1921–1930 construction and retirements separately, with constructions shown as positive bars and retirements shown as negative bars. The net sum of the two bars in the right column corresponds to the net stock addition shown in the left column.

For single-family homes, we observed that the conservation equation generally holds for an entire decade. Each decade’s construction and stock data were summed and the conservation equation was applied to obtain the sum of retirements for each decade. This sum was then redistributed linearly over the decade, yielding an adjusted annual retirement series for each decade. In this manner, annual inconsistencies in the survey data were smoothed over. Fig 2, left, shows the decadal stock conservation for single-family units. The left column of each pair of columns shows the total housing stock of the previous decade, subdivided into the previous year’s stock (solid color) and the net stock additions (light brick pattern). The latter correspond to the number of constructions minus the retirements for that decade. For additional clarity, the right column shows the net stock addition decomposed into the absolute number of constructions (light colored hatch) and retirements (dark colored hatch). The number of constructions is larger than the difference in stock from one decade to the next for most decades. For the 1950’s, 1970’s and 1990’s, however, applying the conservation equation to each decade still revealed decadal stock increases which were not unaccounted for by construction, with the largest inconsistency occurring in the 1970’s.

For multi-family units, the decadal linear smoothing process used for single-family units did not produce consistent results. Here, when the construction data summed over the decade did not account for the increase in stock from one decade to the next, it was assumed that there were no retirements in that decade. The corresponding annual construction data was increased by a value corresponding to the adjustment made to the retirement. In this manner, the stock change from one decade to the next was unaltered, since the difference between constructions and retirements remained unaltered. Decadal stock conservation for multi-family units is shown in Fig 2, right. For some decades, this adjustment approach did not resolve the issue of stock which is unaccounted for, particularly for the 1970’s and 1980’s.

For manufactured homes, because of poor data quality, a decadal approach did not produce consistent results and retirements were estimated annually from the conservation equation. The largest inconsistency occurred in the 1990’s (S1 Fig).

### Survival dynamics and estimation of vintage-disaggregated retirement distributions

The annual retirement total, obtained as described in Section 2.3., aggregates housing units of all vintages. Because housing units belonging to different vintages differ in average floor space, an estimation of floor space requires knowing how many units of each vintage retire every year. Since only partial vintage data are reported by the American Housing Survey, the next step consisted in a disaggregation of the retirement stock into annual vintages for the entire 120-year period. Retirement distributions were adopted for this task. For single-family-and multi-family homes, the probability of retirement of a housing unit occurring after it has reached a certain age was modeled by survival functions; for manufactured homes, linear decay was assumed.

For single-family-and multi-family homes, the probability that retirement of a housing unit occurs *after* the housing unit has reached age *a* is given by the survival function *s(a)*, shown below (Eq 2). This formulation is based on the Commercial Demand Module of the EIA’s National Energy Modelling System, a policy analysis tool which includes a projection of new and surviving commercial buildings as part of its forecast of commercial energy demand [16]. To estimate the number of housing units of each vintage that retire in a certain year, the total aggregated retirement for that year was then distributed over the vintages according to the survival function. The fraction of housing units of a certain age retiring each year was assumed to be the same for all years.

$$s\left(a\right)=\frac{1}{1+{\left(\frac{a}{L}\right)}^{\gamma }}$$ Eq 2

where

*s(a) =* probability that unit retires after age *a* (unitless)

*a =* vintage or age of housing unit (years)

*L* = lifetime, or period of time after construction when half the buildings have retired (years)

*γ* = survival parameter, or rate at which buildings retire near the median expected lifetime *L* (unitless)

The survival function lifetime and survival parameters were adjusted based on calibrations of the resulting estimated vintage stocks with the existing vintage-disaggregated U.S. Census Bureau stock survey data (survival dynamics parameters and graphs are shown in S5 File). We calculated the fraction of stock of each vintage still standing in a particular year, relative to total stock, and chose survival function parameters that minimized the difference between these fractions and the equivalent fractions based on U.S. Census Bureau stock survey data for the 1999–2011 period. In the data provided by the American Housing Survey, as will be mentioned in the discussion about average floor space (Section 2.5), reporting sample sizes in the 1999–2009 surveys are almost twice as large as sample sizes in the 1985–1997 period, so data from the 1999–2011 period was used for the calibration.

For manufactured homes, the disaggregation of the retirement stock into vintages was based on linear decay for each of four vintage periods (pre-1892, 1892–1941, 1942–1981 and 1982–2011), with minimum lifetimes before retirement and a decay rate for each period. This differentiation made it possible to account for data inconsistencies between the periods, and also to base the calibration of the time-series on the more reliable data from the 1982–2011 period.

### Estimation of average floor space

Two sources were initially considered for vintage-disaggregated average floor space data, the American Housing Survey (AHS) and Residential Energy Consumption Survey (RECS). AHS was chosen as the most reliable source for the following reasons. First, AHS sampling sizes are on the order of ten times RECS sampling sizes. While RECS sampled 4,382 households in 2005 and 12,083 in 2009 [17], AHS collected approximately 60,000 samples for an extended period of time, and triple that in 2011 [11]. Second, AHS data have issues associated with self-reporting, while Residential Energy Consumption Survey (RECS) data is measured by surveyors, but overall RECS data collection methodology exhibits more variability over the survey years than the AHS methodology [11]. Third, AHS surveys have been conducted in odd-years since 1985, while RECS surveys have taken place only every three or four years since the 1984 survey.

**Fig 3:**
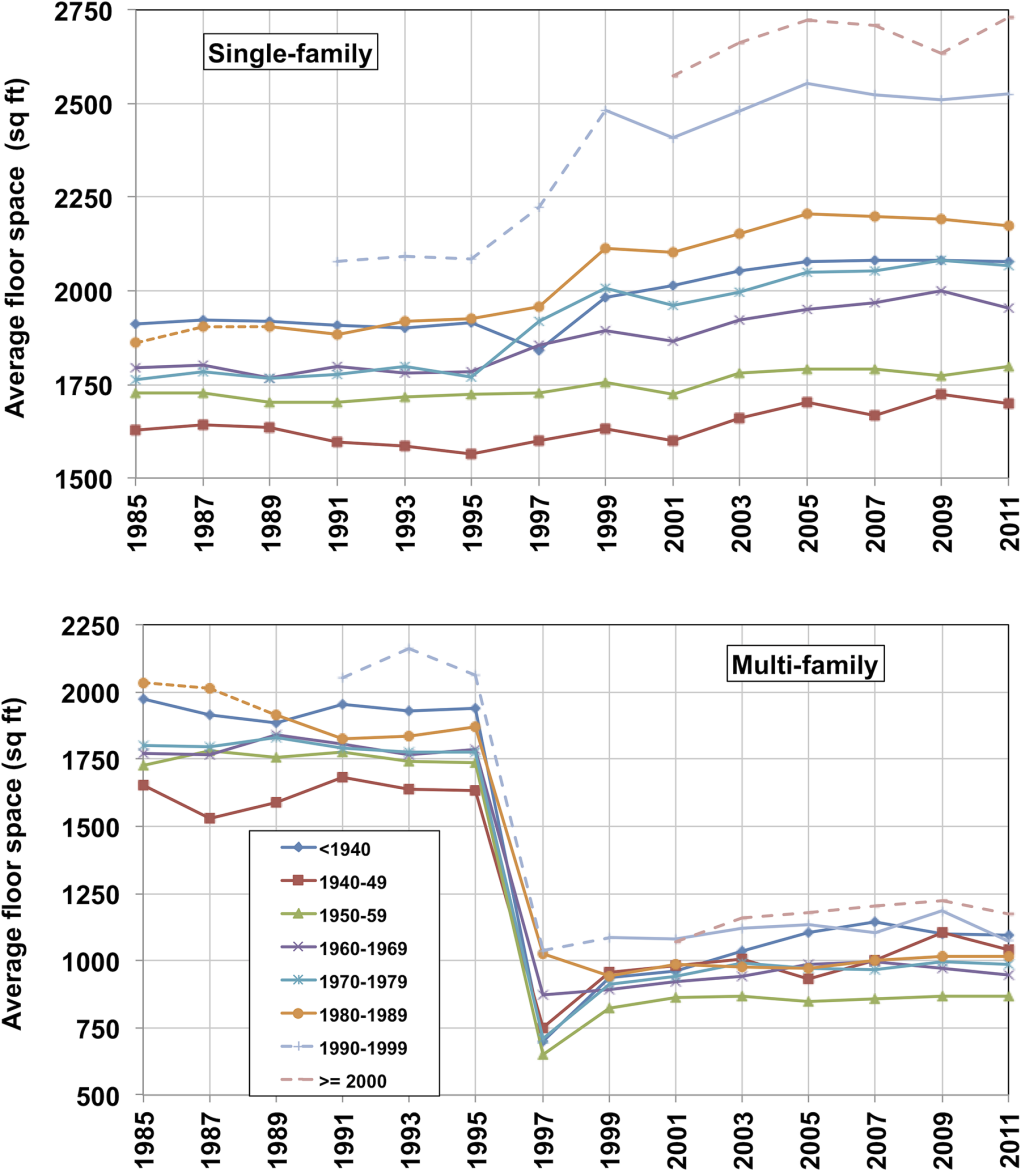
Single-and multi-family average floor space per unit for 8 vintages, from survey years 1985–2011. Dotted lines represent averages computed before vintage period is over. Discontinuities in 1995–1999 are possibly a result of methodology changes. Average floor space for both building types used in this work corresponds to the average of survey year data for 1999–2011. Source: American Housing Survey (S4 File).

As in a recent study [11], this data is aggregated into eight vintage classifications, corresponding to a pre-1940 aggregated vintage and one vintage for every decade since 1940. Fig 3 shows this survey data for single- and multi-family units.

For different survey years, the floor space averages show discontinuities of different degrees for all vintages. The moment of discontinuity is not the same for the different building types: it occurs somewhere between 1995 and 1999 for single-family homes, depending on the vintage, but in 1995–1997 for all vintages of multi-family and manufactured homes. The average floor space data before and after the discontinuity show changes in opposite directions: for single-family homes, the average floor space after the discontinuity is larger than before, while for the other two types the average floor space is approximately 40% smaller after the discontinuity.

The existence of these discontinuities indicates that there have possibly been methodological changes in the survey process. For instance, reporting sample sizes for average floor space in the 1999–2011 surveys are almost twice as large as sample sizes in the 1985–1997 period. Sample sizes increased from approximately 25,000–29,000 observation samples in the 1985–1999 surveys, to 42,000–48,000 samples in the 1999–2009 surveys, to 134,000 samples in 2011, for all housing types and all vintages [18] (S4 File). A choice was therefore made to estimate total floor space based on floor space averages from more recent survey period, after the discontinuities. Table 1 shows the estimated floor space averages for the 1999–2011 survey period.

**Table 1:** Floor space averages. Estimated as average of floor space data from the 1999–2011 period. Source: American Housing Survey (S4 File).

*Based on 1999–2011 data*	<1940	1940–49	1950–59	1960–69	1970–79	1980–89	1990–99	> = 2000
**Single-family**	2,052	1,669	1,773	1,936	2,030	2,162	2,498	2,673
**Multi-family**	1,055	1,004	856	951	967	987	1,112	1,168
**Manufactured homes**	1,209	766	887	921	1,093	1,194	1,459	1,643

As a sensitivity test, the final floor space time-series based on floor space averages before the discontinuities was also estimated. The difference between the two floor space estimates is less than 8% for the entire 120-year period. The averages based on the 1985–1997 period, along with the resulting floor space time-series, are shown in Fig D in S6 File.

There appears to be a trend in increasing average floor space from one survey year to the next after 1999 for some vintages, particularly for single-family units, as can be seen in Fig 3. This trend could be explained by a ‘retirement bias’—the demolition of relatively smaller units—or by floor space additions to existing units, or by other reporting biases. Nonetheless, a choice was made to use a constant average floor space per vintage for all years, due to lack of more detailed information about retirement biases and floor space additions. This choice might have underestimated the trend over time in floor space growth, and would presumably be more important for the older vintages, as additions accumulate over time.

## Results and Discussion

**Fig 4:**
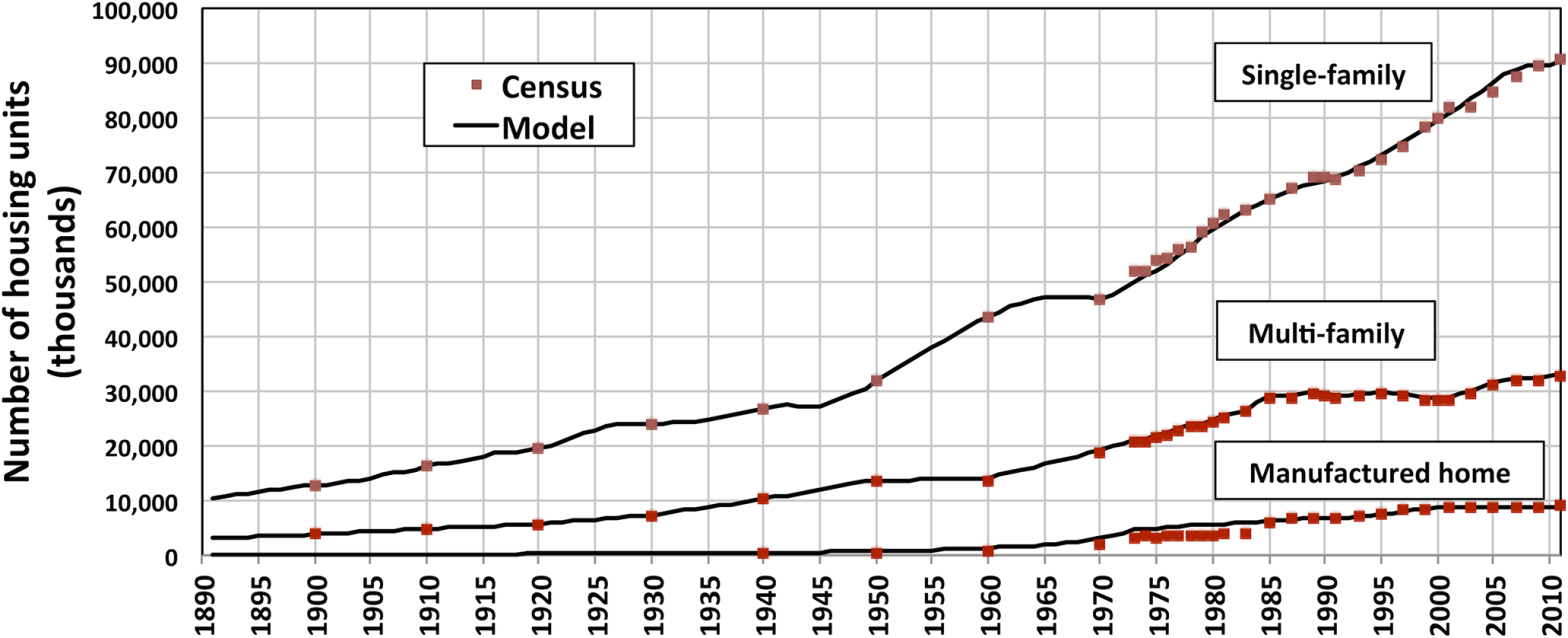
Total U.S. housing stock time-series for 3 building types, 1891–2010. U.S. Census Bureau survey data are shown for comparison. Data include occupied and vacant units.

**Fig 5:**
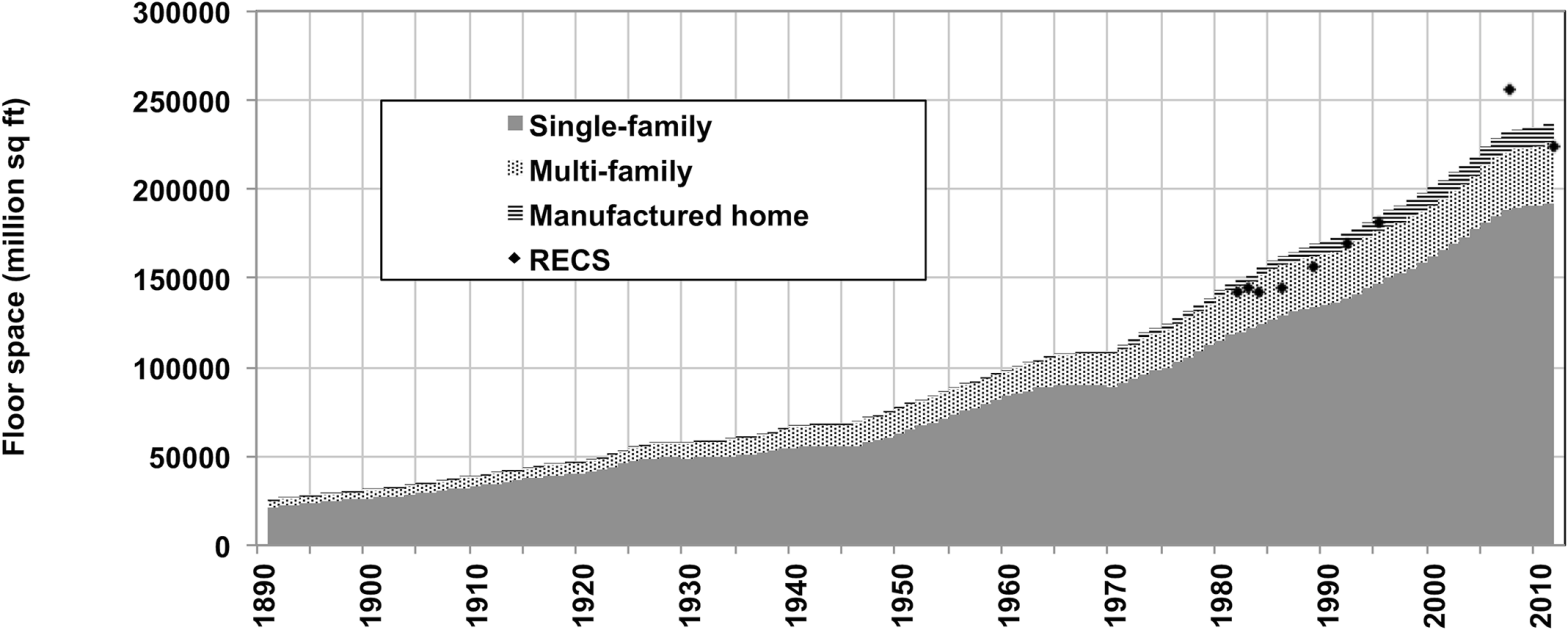
Total U.S. floor space time-series for 3 building types, 1891–2010. RECS data for select years are shown for comparison (RECS data include heated and cooled floor space). Graph in metric units is shown in Fig E in S6 File.

Section 2 described how consistent, long-term and vintage-disaggregated time-series of housing stock were developed from new construction and housing stock survey data. The total annual U.S. floor space for 1891–2010 was then estimated as a product of the housing stock for each vintage and the corresponding estimated floor space per unit average for that vintage. Figs 4 and 5 show the final results.

The number of U.S. homes and their associated total floor space have risen dramatically over the last century. From the end of the 19^th^ to the mid-20^th^ century, a net average of half a million homes was added annually to the country’s stock, corresponding to over 850 million square feet added annually. In the last half century, the net average of homes added annually doubled to one million, which led to a tripling of floor space growth, with 2,700 million square feet added annually, with new construction far exceeding retirements. Over the 1891–2010 period, floor space increased almost tenfold, which corresponds to a doubling of floor space per capita (from approximately 400 to 800 square feet (Fig G in S6 File).

Results show a stock evolution that closely fits the original stock survey data. For single- and multi-family units, which constitute 99% (late 19^th^ C) to 93% (recent decades) of the total stock, the differences between reported stock and model results are less than 10% for all years. Furthermore, in spite of uncertainty in vintage disaggregation data and simplifying assumptions for vintage floor space averages, the final floor space evolution is consistent with historical events (Fig F in S6 File).

**Fig 6:**
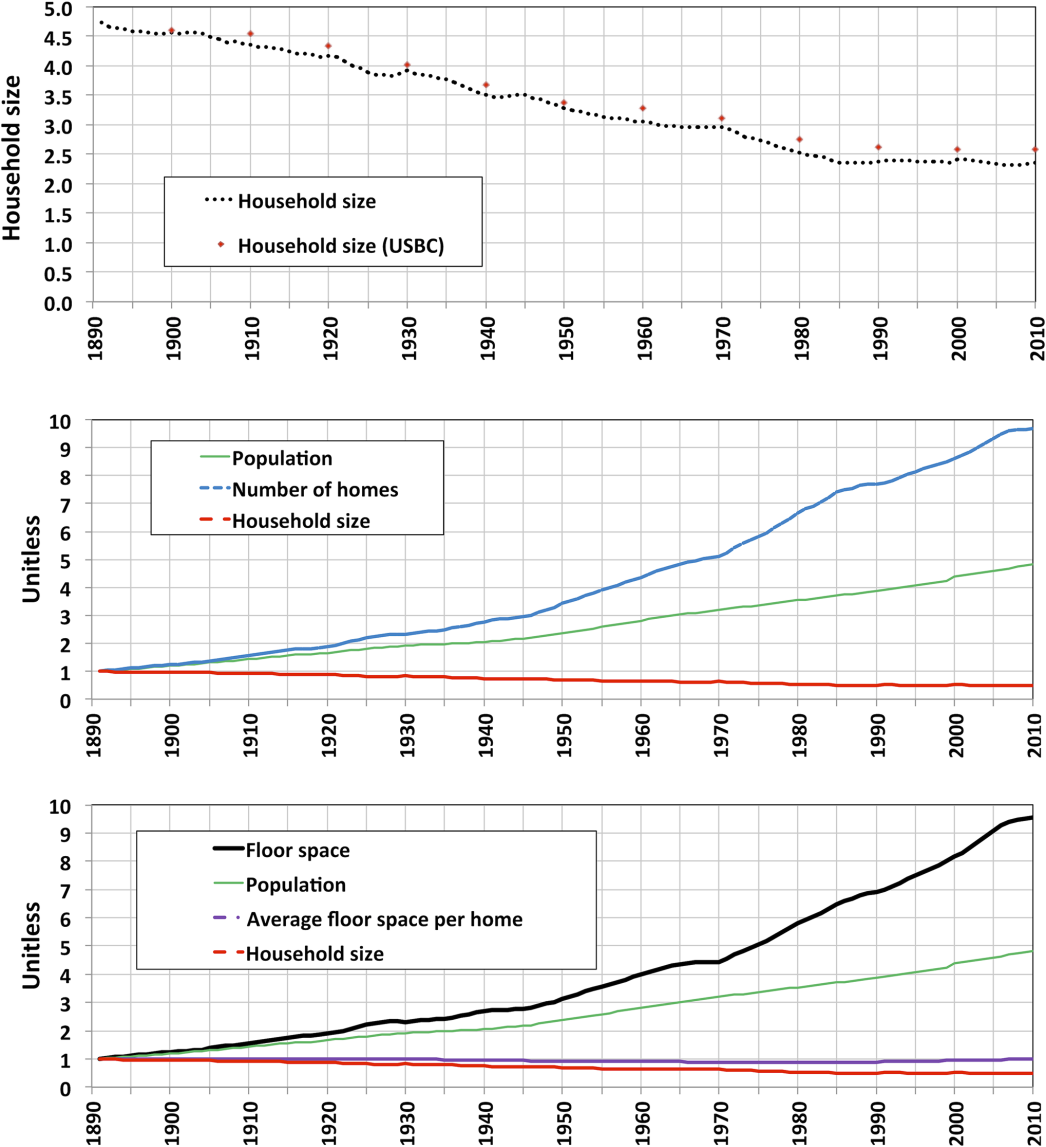
Absolute household size and decompositions of population and floor, 1891–2010. **Top**: absolute household size, derived as the ratio of population and number of units. U.S Census Bureau data are shown for comparison. **Center**: decomposition of population into number of homes and household size. **Bottom**: decomposition of floor space into population, floor space per home and household size. In two lower figures, variables have been normalized to 1 at start of period (1891), so y-axis is unitless. Decomposing household size into the 3 building types was not possible due to insufficient population data per building type.

In order to gain insight on the evolution of floor space, simple decompositions were performed to illustrate the relative evolution of floor space compared to housing stock, population and household size. Between 1891 and 2010, the number of housing units increased by almost ten times, an increase which is similar to the tenfold increase in floor space. But population increased by less than five times in this period, half as much as the increase in floor space and number of units. This can be explained by the role of household size as a major floor space driver. In the period studied, household size was halved (Fig 6, *top*). When household size decreases as population grows, the number of housing units rises more rapidly than population, so floor space was more closely coupled to the rise in number of housing units, rather than to population (Fig 6, lower left).

**Fig 7:**
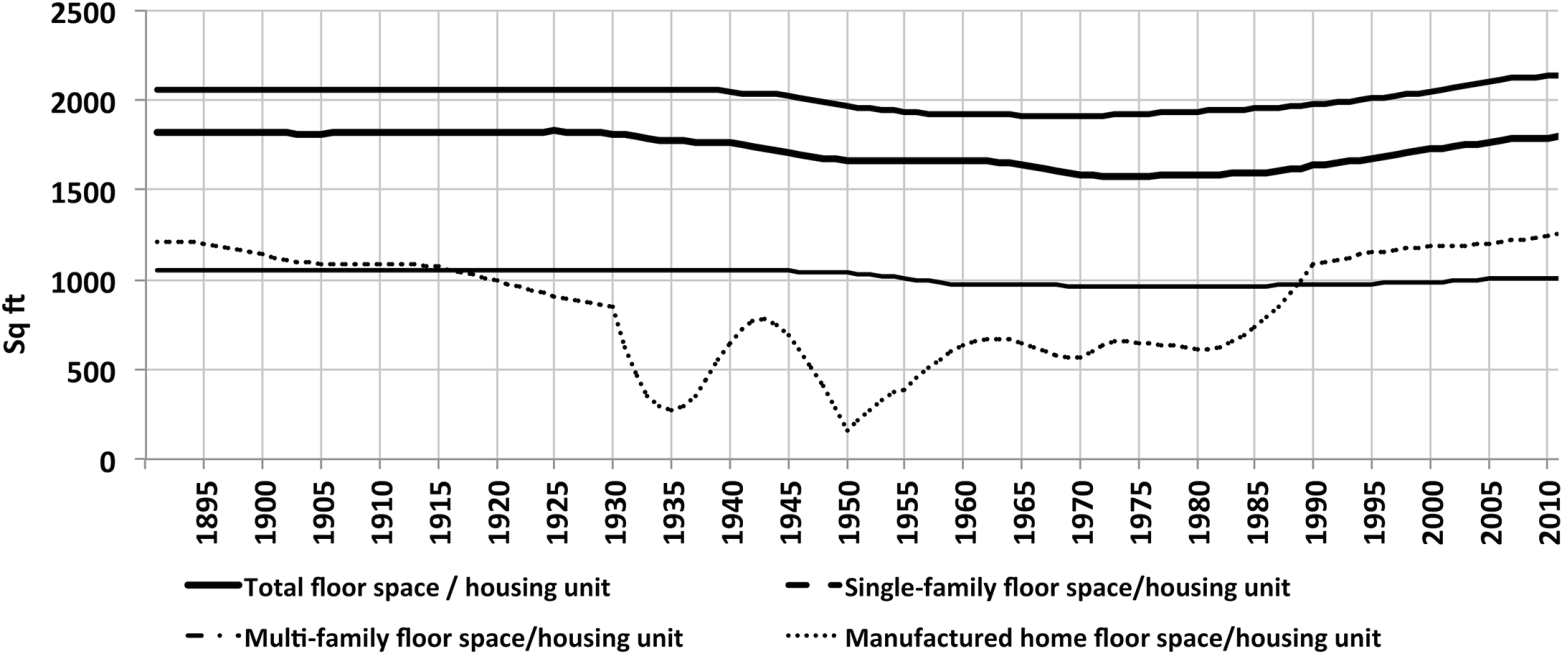
Estimated floor space average by building type, 1891–2010. Ratio of total estimated floor space and housing stock time-series.

Fig 7 shows the estimated aggregated floor space average over the 120-year period, for each building type, calculated as the ratio of the two estimated time–series, total U.S. floor space and housing stock. Average floor space per unit remained approximately constant throughout the period. A slight ‘dip’ occurred in the 1940–2000 period, but the average returned to pre-1940’s values in the 2000’s, and so floor space per unit averages played a relatively minor role in overall floor space evolution over the very long term. This is also evident from Fig 6, lower right, which shows floor space decomposed into population, average floor space and household size. The evolution in overall average floor space mirrors the dynamics observed in the evolution of single-family home average floor space, for this sector constitutes the vast majority of units in the 120-year period (almost 80% in late 19^th^C to almost 70% in recent years) (S2 Fig). In the 1940’s, the addition of a vintage of new homes 20% smaller than the pre-1940’s units caused the ‘dip’ in floor space average. The average recovered as new single-family homes built over the following decades were progressively larger (in the 2000’s, average floor space was 30% larger than that of pre-war years), compensating the retirement of the pre-1940 vintage (the stock in this vintage decreases from almost 80% at the end of the 1940’s to slightly less than 25% in 2000).

GDP has traditionally been considered one of the major drivers of floor space growth, since housing costs are a significant fraction of income. Average consumer expenditure on housing from 1986 through 2010 increased from 29.7% to 33.2% for homeowners and from 32.9% to 38.4% for renters [19]. In 2011, more than one in four renters and one in five owners experienced a “severe housing cost burden”, signifying they spent at least half their income solely on housing costs [20]. Even though it is beyond the scope of this work to examine the role of GDP as a driver of floor space, it is instructive to compare the evolution of floor space per capita and GDP per capita. As a result of the difficulty in obtaining floor space datasets that are coherent across regions and over time, some studies have been forced to resort to estimations of floor space based on indicators such as population or gross domestic product (GDP), without first establishing causality [21]. One recent study, for instance, constructs building energy use datasets for 2010, for 10 regions in the world, based on 2005 floor space data. The authors use population to scale 2005 residential floor space data to 2010 and GDP to scale commercial floor space data to that same year [21].

**Fig 8:**
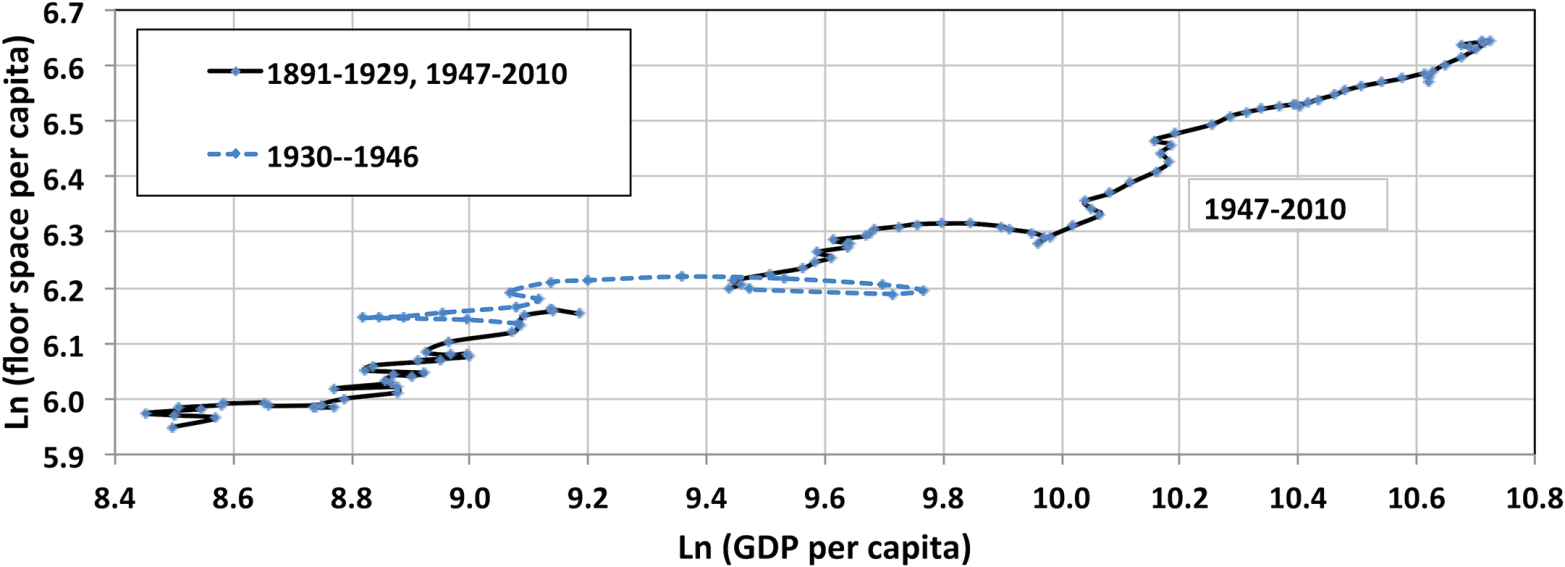
Log-linear relationship between floor space per capita and GDP per capita. Sources for GDP: for 1969–2010, a compilation of historical data by the Unites States Department of Agriculture was used [22]; to extend data back to 1890, the Maddison Project was used [23].

We find that the relationship between floor space per capita and GDP per capita is remarkably similar over the long-term, as can be seen from their log-linear relationship, shown in Fig 8, with a substantial disruption during the great depression (see also Fig H in S6 File for more detailed comparison). We emphasize that this does not imply causality; there are certainty substantial commonalities in the drivers of floor space and GDP. As discussed above, for example, decreasing household size was a major driver of historical US floor space trends. Some of the demographic and social factors behind the trend in household size are likely to have also impacted GDP.

It should be noted that it was beyond the scope of this work to address the many potential macro-economic drivers of housing stock and floor space, such as for instance, disposable income, inflation and unemployment. Housing market variables, such as mortgages and housing prices, are likely to be particularly important. Also likely to be important are social and demographic factors that influence household size. These factors, and other relevant missing variables, should be considered in future formal analyses of floor space drivers.

**Fig 9:**
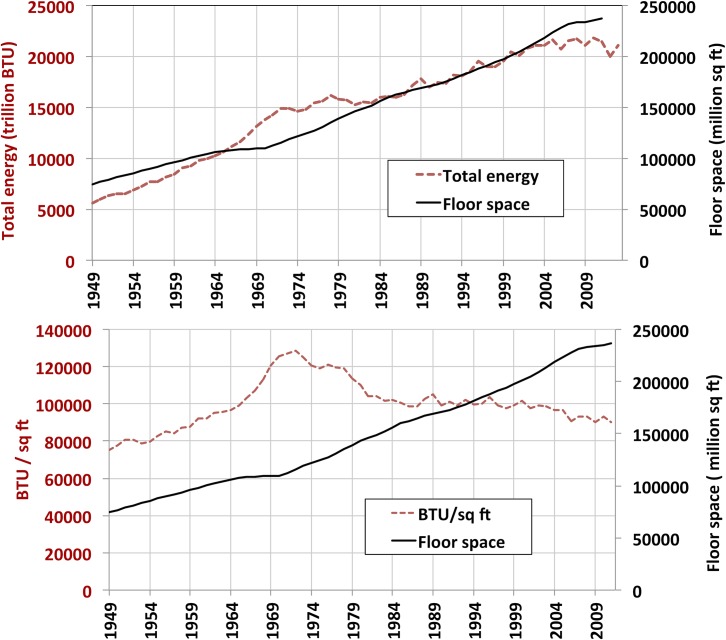
Total energy consumption and total energy consumption per square foot, compared to floor space. Total energy includes all end-use fossil and renewable sources, electricity, electric conversion and distribution losses. Source for energy: EIA Monthly Energy Review [1].

In a final comparison, the joint evolution of floor space and primary energy consumption for the second half of the century was briefly examined (Fig 9). From the post-WWII period up until the first oil crisis, energy consumption increased at a faster rate than floor space, especially in the late 1960s. From the oil crisis until the early 1980s, the rate of energy consumption decreased relative to floor space growth. From the 1980s to the beginning of the 2000s, energy consumption and floor space grew at the same rate. In the last few years of this period, energy per square foot first decreased and then stabilized. Future trends are, of course, unknown, but the historical perspective provided by this work provides valuable context for examination of past trends.

## Conclusion and Future Research

Our results show that over the last 120 years floor space and housing stock in the U.S. increased approximately tenfold, while population increased approximately fivefold and household size decreased by a dramatic 50%. Average floor space has remained approximately constant. But can these long-term trends be expected to continue into the future? The interplay between the evolution of population, household size, average floor space growth, as well as other factors, such as the construction rate of single-family homes and the retirement of older smaller vintages, could become significant in future dynamics.

In order to look ahead, it is important to distinguish between short-and long-term trends. The 2008 housing crisis is a recent event from which the housing market is still recovering, so trends in the last decade may not be representative of future developments. Taking the last 30 years into account, however, allows for a better insight into the near future. It is particularly instructive to examine the two factors that have evolved differently over the last three decades, compared to their previous evolution, namely household size and average floor space.

Decreasing household size is a trend with roots in a combination of factors: increased national income, increased mobility, demographic factors such as the aging of the population and the proportion of young adults who are potential homebuyers, and cultural factors such as changing family structures (ex. the increase of the median age at first marriage, family size and the overall decline in the number of married adults, [24]. These factors could contribute to a further long-term decrease of household size, but compared to the decrease in household size since the late 19^th^C, household size in the U.S. has been decreasing at a slower pace since the 1980’s. In 2007–2010 there was a slight increase from 2.31 to 2.34 persons per household (Fig 6, *top*), which could either be immediate consequence of the housing crisis, or could indicate a more fundamental change in the long-term trends. A slow economic recovery, with still relatively high unemployment, a rising student debt and the difficulty of obtaining mortgage credits, are all factors that may contribute to a slower decrease or an increase in household size, as young adults are less able to move out of their parents’ homes [25].

In the last 30 years floor space averages have been increasing, as older post-1940 vintages consisting of smaller units, such as those built in the post-war “baby boom” years progressively retire, while larger units are added to the stock. New single family homes built in the first decade of the 21st century averaged 2,673 square feet, while those built in the 1980’s averaged 2,162 square feet (see Table 1). The overall average floor space for units of all building types increased by 13% in the last 30 years, reaching almost 1,800 square feet per unit in 2010 (see Fig 9).

These two factors–household size and floor space averages—have the potential to drive floor space in opposite directions. The observed increase in average floor space is a clear trend, which might only be attenuated if the rate of construction of single-family homes or retirement of older units also slows down. On the other hand, if the tendency for household size to decrease more slowly or increase is indeed a new trend, then both the number of housing units and floor space might accompany population growth more closely than has been the case in the past.

The long-term floor space series presented here can be improved by addressing in more depth the inconsistency and incompleteness issues found in the survey data. A further understanding of methodological issues that affect the quality of the data can improve the choice of data used to develop the time-series. An effort can be made to collect data on renovations, such as floor space additions to existing houses, and on retirement biases, making it possible to disaggregate retirement by floor space as well as by vintage. Data in this study have taken both occupied and vacant units into account, so an attempt can be made to use partial data to estimate occupied floor space, as well as heated and cooled floor space. Household size data can be improved by disaggregating by type of building. From a broader perspective, regional data, including metropolitan data, can be used to better estimate national totals. In 2011, residential buildings were responsible for 22% of total U.S. energy consumption, while commercial buildings did not lag far behind, at 19%, so developing floor space time-series for the commercial sector is also highly relevant.

## Supporting Information

S1 Fig Results: Annual retirement time-series. (DOCX)

S2 Fig Results: Percentage of building types in total stock. (DOCX)

S1 File Terminology and basic assumption. (DOCX)

S2 File Survey data and time-series: New construction. (DOCX)

S3 File Survey data and time-series: Housing stock. (DOCX)

S4 File Survey data: average floor space. (DOCX)

S5 File Results: Retirement distributions and survival dynamics. (DOCX)

S6 File Results: Floor space time-series. (DOCX)

S7 File Calibration and validation. (DOCX)
