# Supplementary material for: 120 Years of U.S. Residential Housing Stock and Floor Space
Source: PLoS One. 2015 Aug 11;10(8):e0134135. doi: 10.1371/journal.pone.0134135 (PMC4532357; doi:10.1371/journal.pone.0134135)
Supplement: S1 Fig — (DOCX) [file pone.0134135.s001.docx]

**S1 Fig. Results: Annual retirement time-series**

Annual apparent retirements for 3 building types, 1891-2010. calculated according to conservation equation and retirements used by model (SF: sum of retirements for each decade is linearly smoothed - MF: retirements are set to zero for decades when decadal sum is negative and compensated in construction - MH: retirements used in model are those calculated by conservation equation.
