# Supplementary material for: 120 Years of U.S. Residential Housing Stock and Floor Space
Source: PLoS One. 2015 Aug 11;10(8):e0134135. doi: 10.1371/journal.pone.0134135 (PMC4532357; doi:10.1371/journal.pone.0134135)
Supplement: S2 Fig — (DOCX) [file pone.0134135.s002.docx]

# S2 Fig. Results: Percentage of building types in total stock

Stock time-series showing percentage of single-family, multi-family and manufactured relative to total stock, 1891-2010. Calculated from estimated housing stock time-series.
