# Supplementary material for: 120 Years of U.S. Residential Housing Stock and Floor Space
Source: PLoS One. 2015 Aug 11;10(8):e0134135. doi: 10.1371/journal.pone.0134135 (PMC4532357; doi:10.1371/journal.pone.0134135)
Supplement: S1 File — (DOCX) [file pone.0134135.s003.docx]

# S1 File. Terminology and basic assumptions

- USCB: U.S. Census Bureau
- HUD: U.S. Department of Housing and Urban Development
- EIA: U.S. Energy Information Administration
- AHS or AmHS: American Housing Survey
- RECS: Residential Energy Consumption Surveys
- Floor space: measurement of the two-dimensional area of a housing unit enclosed from the weather. In the USCB data used here, floor space only accounts for finished areas, including basements and attics, but does not include garages or porches. In RECS, floor space includes basements, and attics and attached garages only if they are heated, cooled or finished.

Sources:

- - U.S. Census Bureau. New Residential Construction, Definitions. n.d.
  - Energy Information Administration. RECS 2009, Where does RECS square footage data come from? EIA; 2012.
- Building types: SF = single-family homes; MF= multi-family units; MH= manufactured or mobile homes.
- Construction: number of new buildings constructed in a certain year; does not take additions to existing houses into account
- Stock: number of housing units of a type of building standing in a certain year
- Annual retirements: the total number of housing units that retire in a certain year, aggregated over all vintages.
- Retirement distribution: the distribution of annual retirement over all vintages.
- Vintage: classification of buildings into periods based on when they were constructed. In this study, eight vintages are used.
- Depending on the classifications used in the source, SF units are assumed to be equivalent to the *1-unit* or to the sum of *single-family detached* and *single-family attached* homes; MF units are assumed to be equivalent to sum of buildings with *2-units* and *3-unit or more*, or to sum of buildings with *2-4 units* and *5 or more units*.
- Stock data take into account vacant and occupied units.
- Floor space averages account for all residential floor space: heated, cooled, non-heated and non-cooled areas.
- Whenever data are missing, linear interpolations are made, unless otherwise noted.
- Disaggregation: using a coefficient to calculate constituent parts based on an aggregated total.
- Adjustment = multiplying by a coefficient. Coefficients are selected based on calibration of the model.
