# Supplementary material for: 120 Years of U.S. Residential Housing Stock and Floor Space
Source: PLoS One. 2015 Aug 11;10(8):e0134135. doi: 10.1371/journal.pone.0134135 (PMC4532357; doi:10.1371/journal.pone.0134135)
Supplement: S2 File — (DOCX) [file pone.0134135.s004.docx]

# S2 File. Survey data and time-series: New construction

## Original sources

Source for 1889-1970

Historical Statistics of the United States, Colonial Times to 1970 - Part 2 n.d.; Series N 156-169

Source for 1971-2013

U.S. Census Bureau, New Residential Construction

Source for 1959-2014

U.S. Census Bureau, Manufactured Homes Survey in Business and Industry: Time Series / Trend Charts n.d.

## Assumptions

The construction time-series was developed based on different data sources, which were adjusted and combined to produce a consistent time-series. Some data for construction do not include farms. In these cases, the following coefficients were used to derive farm construction:

Table A. Farm coefficients for construction, 1889 through 1950s

| **1889 and 1890s** | **1900s** | **1910s** | **1920s** | **1930s** | **1940s** | **1950s** |
| --- | --- | --- | --- | --- | --- | --- |
| 1.80 | 1.70 | 2.00 | 2.00 | 2.00 | 1.05 | 1.03 |

The following coefficients were used to disaggregate farm constructions:

Table B. Building type disaggregation coefficients for construction, pre-1900 and 1945-1958

|  | **1-unit** | **2-unit** | **3 or more units** |
| --- | --- | --- | --- |
| **Pre-1900** | 0.65 | 0.14 | 0.21 |
| **1945-1958** | 0.80 | 0.06 | 0.14 |

#### 1889-1899

Aggregated annual construction *without MH and* *without farms* is available for these years. For 1-units, 2-units and 3 or more units, coefficients as listed above were applied to include farms and disaggregate. Ex: Original data for 1889 consist of 342,000 units. After farm adjustment and disaggregation, we obtained 400,140 1-units, 47,880 2-units and 71,820 3 or more units.

Data exist for MH shipments in 1947-1970. We take the percentage of MH relative to 1-units in 1947 (6%) and apply that coefficient to the 1-units to obtain the MH data for this period. Ex: Derived data for 1-units in 1889 consist of 400,140. After applying coefficient, we obtain 22,541 MHs.

#### 1900-1944

Disaggregated annual construction *without MH and* *without farms* is available for 1900-1944. Coefficients as listed above were applied to include farms. Ex: Original data for 1900 buildings consist of 123,000 1-units. After farm adjustment, we obtained 1-209,100 1-units.

MH data for this period was obtained as in previous period. Ex: Derived data in 1900 consist of 209,100 1-units. After applying coefficient, we obtained 11,779 MHs.

#### 1945-1946

Aggregated annual construction *without MH and* *without farms*,is available for these years. Coefficients as listed above were applied to include farms and disaggregate. MH data for this period was obtained as in previous period.

#### 1947-1958

Aggregated annual construction *without farms* is available for 1900-1944. Coefficients as listed above were applied to include farms and disaggregate. MH shipment data are available for this period.

#### 1959-1962

Disaggregated annual construction *without farms*, is available for these years. Coefficients as listed above were applied to include farms. MH shipment data are available for this period

#### 1963-1970

Disaggregated annual construction *with farms* is available for these years. Construction data are also available for 1968-2013 from the USCB. Statistical Abstract census data [5] overlap for 1968-1970 but the two datasets are not consistent. For SF, differences are less than 5%, but for MF they differ by 40% for 1968, 16% for 1969 and 6% for 1970. We chose to use the Historical Statistics of the United States, Colonial Times to 1970, for these 3 years, as values are higher and this minimizes the increase from 1970 to 1971. MH shipment data are available for this period.

#### 1971-2010

For 1971-2010, Census data are available for SF, MF and MH.

## Time-series

Table C. Construction time-series for 3 building types, 1891-2010
