# Supplementary material for: 120 Years of U.S. Residential Housing Stock and Floor Space
Source: PLoS One. 2015 Aug 11;10(8):e0134135. doi: 10.1371/journal.pone.0134135 (PMC4532357; doi:10.1371/journal.pone.0134135)
Supplement: S3 File — (DOCX) [file pone.0134135.s005.docx]

# S3 File. Survey data and time-series: Housing stock

## Original sources

Source for 1900-2002

- USCB, Statistical Abstracts of the U.S.: 2003. 2003. (Table No. HS-12. Households by Type and Size: 1900 to 2002)

Source for 1940-2000

- USCB Census of Housing, Historical Census of Housing Tables - Units in Structure n.d.

USCB Decennial Census 2000, Part 2, PHC-2 , Tables 9 and 10

Source for 1973-1989 (odd years)

U.S. Department of Housing and Urban Development. Housing Trends in the U.S., 1973 to 1989. n.d.

Source for 1973-1983

- USCB Annual Housing Survey, Tables in h150 series

## Assumptions

The stock time-series were developed based on different data sources, which were combined to produce a consistent time-series. The time-series are shown in Table 5.

#### 1889-1930

Aggregated stock for decadal years is available for 1900-2002 from the USCB Statistical Abstracts. Disaggregated stock is available for 1940-2000 from the USCB Census of Housing. When data for the overlapping years 1940-2000 were compared, stock as reported by the Census of Housing was on the average 9% larger. So we adjusted the Statistical Abstract [5] aggregate stock by this percentage for 1900-1930 to obtain consistent set of stock data for 1900-2000.

To disaggregate stock for 1900-1930, for lack of a better option, a coefficient was derived based on the proportion of each type of building in construction data and was applied to the stock data. The estimated fractions of type of building relative to all buildings obtained, based on construction, were the following:

Table A. Building type disaggregation coefficient for stock, 1890-1930

|  | **SF** | **MF** | **MH** |
| --- | --- | --- | --- |
| **1889-1930** | 0.74 | 0.22 | 0.04 |

Ex: In 1900 there are 15,964,000 units of all types. Applying the 9% coefficient, this becomes 17,362,768. Disaggregating with construction-based coefficient, we obtained 12,801,301 SF units, 3,840,349 MF units and 721,119 MH units.

#### 1940-1970

Disaggregated stock is available from the Census of Housing.

#### 1973-1989

The USCB ‘h150 series’ [8] for 1973-1983 provides annual data (with the exception of 1982, for which there are no data), while the HUD 1973-1989 Housing Trends provide data only for odd years, but for a longer period. These datasets differ by less than 2%, due to differences in the SF attached units. We chose to use the longer HUD dataset for the odd years, and for even years up to 1978 we used the USCB series with an adjustment to the SF attached data. Stock for even years 1980-1988 was interpolated.

There are several inconsistencies in the data for the 1970s and 1980s which we did not attempt to resolve. First, the HUD and USCB sources use different sample weights in the 1970s (see 1973-1989 Housing Trends). Second, in 1985 there was a change in sampling methodology, based on use of the 1980 Decennial Census data. At this time, the Annual Housing Survey became the American Housing Survey. As a result, the 1973-1983 data are not consistent with data reported after 1985.

#### 1991-2011

The data for odd years are available from the AHS.

For 1997, 1999, 2001, 2003, 2005, 2007, 2009, 2011:

- http://www.census.gov/housing/ahs/data/national.html

For 1985, 1987, 1989, 1991, 1993, 1995:

- <http://www.census.gov/housing/ahs/publications/historical.html>

For 1973, 1977, 1979, 1981, 1983:

- http://www.census.gov/housing/ahs/publications/h150.html

## Time-series - The stock time-series shown were derived based on the original survey data.

Table B. Data source for number of housing units for 3 building types, 1891-2010
