# Supplementary material for: 120 Years of U.S. Residential Housing Stock and Floor Space
Source: PLoS One. 2015 Aug 11;10(8):e0134135. doi: 10.1371/journal.pone.0134135 (PMC4532357; doi:10.1371/journal.pone.0134135)
Supplement: S4 File — (DOCX) [file pone.0134135.s006.docx]

# S4 File. Survey data: average floor space

## AHS micro-data

This section summarizes the micro-data reported by AHS. The average area of floor space data used to calculate the floor space time-series are derived from these datasets, as are the vintage-disaggregated stock data used for the calibration. Tables A-G summarize average floor space, number of units and corresponding sample sizes as reported in the AHS micro-data, for biennial survey years in the 1999-2011 period, disaggregated over eight vintages, for the three building types. Tables A-G only show AHS data for 1999 onwards, but data for 1985-1997 can be found in AHS micro-data. Tables H, I and J show the average floor space data derived from the micro-data presented in Tables A-G.

Table A. American Housing Survey micro-data for 1999.

Table B. American Housing Survey micro-data for 2001.

Table C. American Housing Survey micro-data for 2003.

Table D. American Housing Survey micro-data for 2005.

Table E. American Housing Survey micro-data for 2007.

Table F. American Housing Survey micro-data for 2009.

Table G. American Housing Survey micro-data for 2011.

**Average floor space derived from micro-data**

The values in Tables H-J were calculated from the AHS survey data given in Tables A-G. We used AHS floor space averages for detached and attached units weighted by the AHS survey number of units.

Table H. AHS survey data: single-family average floor space.

Table I. AHS survey data: multi-family average floor space.

Table J. AHS survey data: manufactured homes average floor space.

**Investigating the 1995-1999 discontinuity in the AHS micro-data**

Table K summarizes the percentage difference in the AHS average floor space survey data in the discontinuity period for each vintage. The largest discontinuity occurs for multi-family and manufactured homes. It should be noted the survey data for manufactured homes are based on a small number of observations, in the few hundreds, for survey years up to 1995, and that after 1995 this sample size is further reduced, to less than 70, including very small samples sizes, such as 9 for the 1940-49 vintage in the 2007 survey.

In order to smooth out this uncertainty, averages used here were based on a recent survey period, (1999-2011) where variations are smaller than the discontinuity variations, and for which samples represent a higher percentage of the total stock than for earlier survey years.

Table K. Percentage difference in average floor space survey data due to discontinuity. Comparison is made between 1997 and 1999 for single-family homes and between 1995 and 1997 for multi-family and manufactured homes, for 7 vintages.

**Investigating the pre-1940 vintages**

In order to check the validity of using one single floor space average for the pre-1940 vintage, we disaggregated the pre-1940 vintage group for 2009 into 3 sub-vintages for pre-1919, 1920-29 and 1930-39. The micro-data do provide some data for pre-1919, 1920-29 and 1930-39 vintages, but floor space averages for these sub-vintages do not differ much from the aggregated pre-1940 floor space average. Differences were no larger than 7%, not significant enough to justify disaggregation of pre-1940 floor space averages into further sub-vintages.

Table L. 2009 average floor space disaggregation of pre-1940 vintage into pre-1919, 1920-29 and 1930-39 vintages (with single-family disaggregated into attached and detached)

Table M. 2009 average floor space disaggregation of pre-1940 vintage into pre-1919, 1920-29 and 1930-39 vintages (with single-family aggregated into one type of unit)
