# Supplementary material for: 120 Years of U.S. Residential Housing Stock and Floor Space
Source: PLoS One. 2015 Aug 11;10(8):e0134135. doi: 10.1371/journal.pone.0134135 (PMC4532357; doi:10.1371/journal.pone.0134135)
Supplement: S5 File — (DOCX) [file pone.0134135.s007.docx]

# S5 File. Results: Retirement distributions and survival dynamics

Figure A. Survival rates and retirement distributions for single- and multi-family units

Table A. Parameters used in retirement distribution of single-family units

|  | Single-family units | Multi-family units |
| --- | --- | --- |
| Survival parameter γ | 62.8 | 35.2 |
| Lifetime | 2.3 | 8.1 |

Table B. Parameters used in retirement distribution of manufactured homes

| **Manufactured homes** | |
| --- | --- |
| **Minimum lifetime before retirement (years)** | 2 |
| **% retiring relative to previous year:** |  |
| **Pre-1892** | 1.0% |
| **1892-1941** | 6.7% |
| **1942-1981** | 2.5% |
| **1982-2011** | 0.3% |
