# Supplementary material for: 120 Years of U.S. Residential Housing Stock and Floor Space
Source: PLoS One. 2015 Aug 11;10(8):e0134135. doi: 10.1371/journal.pone.0134135 (PMC4532357; doi:10.1371/journal.pone.0134135)
Supplement: S6 File — (DOCX) [file pone.0134135.s008.docx]

# S6 File. Results: Floor space time-series

## Floor space based on two floor space averages

This section shows floor space results for floor space averages based on 1985-1997 and on 1999-2011 survey data periods.

Table A. Floor space averages used in floor space time-series based on 1985-1997 period, for three building types and eight vintages

| ***Based on 1985-1997 data*** | **<1940** | **1940-49** | **1950-59** | **1960-69** | **1970-79** | **1980-89** | **1990-99** | **>= 2000** |
| --- | --- | --- | --- | --- | --- | --- | --- | --- |
| **Single-family** | 1,902 | 1,607 | 1,718 | 1,797 | 1,796 | 1,908 | 2,120 | 2,673 |
| **Multi-family** | 1,756 | 1,497 | 1,595 | 1,658 | 1,640 | 1,788 | 1,829 | 1,168 |
| **Manufactured homes** | 1,842 | 1,492 | 1,594 | 1,616 | 1,649 | 1,734 | 1,883 | 1,643 |

Figure A. Floor space for single-family homes based on 2 different floor space averages (1985-1997 and 1999-2011)

Figure B. Floor space for multi-family homes based on 2 different floor space averages, for 1985-1997 and 1999-2011 periods

Figure C. Floor space for manufactured homes based on 2 different floor space averages: for 1985-1997 and 1999-2011 periods

Figure D. Floor space for all 3 building types based on 2 different floor space averages (1985-1997 and 1999-2011)

## Floor space in metric units

Figure E. Floor space in metric units. *Top*: Floor space time series for 3 building types, 1891-2010. *Middle*: Average floor space per capita, for all building types, 1891-2010. *Bottom*: Average floor space per unit for 3 building types and for all building types, 1891-2010.

## Floor space rate of change

The floor space rate of change shows housing cycles over the 120-year period. Five approximate periods characterized by cycles consisting first of accelerating growth and then decelerating growth can be discerned: 1918-1929, 1930-1944, 1945-1966, 1967-1990 and 1991-2009. The collapse due to the Depression is the only moment in the period when the rate of change becomes negative.

Figure F. Floor space rate of change showing housing cycles, 1891-2010

##

## Average floor space per capita

The time-series for average floor space per capita was obtained as a ratio of total U.S. floor space (for all housing units) and population.

Figure G. Floor space per capita, 1981-2010

## Comparing the evolutions of floor space per capita with GDP per capita

In this comparison exercise, we were not investigating causality between floor space per capita and GDP per capita. We assumed a simple exponential relationship for three periods: the pre-depression period (1891-1929), the post-war period (1947-2010) and the entire period:

|  |  |
| --- | --- |

$$FS_{cap}=\beta{{GDP}_{cap}}^{\alpha}$$

Equation A

We chose an exponential model because it approximates the estimated relationship between the floor space time-series developed in this work and GDP per capita. This model also captures an important aspect of the floor space – GDP relationship, namely that the floor space to GDP ratio exhibits a decreasing trend, with a slightly steeper decrease in their ratio in the period before than after the depression and World War II. The results are shown below, along with standard errors (SE) for each regression period.

**Figure H. Regression for floor per capita and GDP per capita.** The regression is based on housing stock conservation equation described in the main text. Also shown are slopes and standard errors (SE) for each regression period. No causality was intended in this comparison.

Sources for GDP: for 1969 -2010, a compilation of historical data by the United States Department of Agriculture was used to extend data back to 1890. Sources:

- United States Department of Agriculture. Economic Research Service International Macroeconomic Data Set. n.d.
- Bolt J, van Zanden JL. The Maddison Project: collaborative research on historical national accounts: The Maddison Project. Econ Hist Rev 2014. doi:10.1111/1468-0289.12032.

It should be noted that any further analyses of this relationship should take into account the fact that GDP data is more uncertain going back in time. Also, since the GDP sources are a compilation of various available datasets, changes in GDP measures over time should be taken into consideration in any future work aiming at a formal analysis of the relationship between GDP and floor space or housing stock.

# 
