# Supplementary material for: 120 Years of U.S. Residential Housing Stock and Floor Space
Source: PLoS One. 2015 Aug 11;10(8):e0134135. doi: 10.1371/journal.pone.0134135 (PMC4532357; doi:10.1371/journal.pone.0134135)
Supplement: S7 File — (DOCX) [file pone.0134135.s009.docx]

# S7 File. Calibration and validation

Table A shows the estimated vintage-disaggregated stock and the corresponding survey data selected from the AHS micro-data for single-family homes (the original AHS micro-data are shown in S4). Figure A shows these two sets of data for single-family homes in graphical format, including the differences between them, for comparison. As an example, Figure B shows the comparisons for 2009 for single-family homes, in percentage format.

The calibration aimed at minimizing the percentage differences for vintage years where differences exceeded 10%. The difference between the study‘s total stock and the AHS total stock for survey years 1999 to 2011 for all building types was less than 3% and for survey year 1985 the difference was less than 8%. Differences for vintages had more variation, dependent on the quality of the data. For single-families, for 1999-2011 survey years, the most significant differences are an overestimation of the pre-1940’s vintage, and an underestimation of the 1970’s vintage, both by about 5%.

Table A. Number of single-family units in estimates (top) and based on AHS survey data (bottom), disaggregated over 8 vintages, for survey years 1985 and odd years 1999-2011.

Figure A. Number of single-family units for odd years in 1999-2011, for study estimates and AHS survey data, including differences, disaggregated by 8 vintages.

Figure B. Comparison of number of single-family units in 2009 based on AHS survey data and study estimates. Percentages refer to fraction of units in a vintage relative to entire 2009 single-family stock.
